# Impact of chronic systolic heart failure on lung structure–function relationships in large airways

**DOI:** 10.14814/phy2.12867

**Published:** 2016-07-14

**Authors:** Steven C. Chase, Courtney M. Wheatley, Lyle J. Olson, Kenneth C. Beck, Robert J. Wentz, Eric M. Snyder, Bryan J. Taylor, Bruce D. Johnson

**Affiliations:** ^1^ Division of Cardiovascular DiseasesMayo ClinicRochesterMinnesota

**Keywords:** Airway walls, airways, congestion, CT, edema

## Abstract

Heart failure (HF) is often associated with pulmonary congestion, reduced lung function, abnormal gas exchange, and dyspnea. We tested whether pulmonary congestion is associated with expanded vascular beds or an actual increase in extravascular lung water (EVLW) and how airway caliber is affected in stable HF. Subsequently we assessed the influence of an inhaled short acting beta agonist (SABA). Thirty‐one HF (7F; age, 62 ± 11 years; ht. 175 ± 9 cm; wt. 91 ± 17 kg; LVEF, 28 ± 15%) and 29 controls (11F; age; 56 ± 11 years; ht. 174 ± 8 cm; wt. 77 ± 14 kg) completed the study. Subjects performed PFTs and a chest computed tomography (CT) scan before and after SABA. CT measures of attenuation, skew, and kurtosis were obtained from areas of lung tissue to assess EVLW. Airway luminal areas and wall thicknesses were also measured**.** CT tissue density suggested increased EVLW in HF without differences in the ratio of airway wall thickness to luminal area or luminal area to TLC (skew: 2.85 ± 1.08 vs. 2.11 ± 0.79, *P* < 0.01; Kurtosis: 15.5 ± 9.5 vs. 9.3 ± 5.5 *P* < 0.01; control vs. HF). PFTs were decreased in HF at baseline (% predicted FVC:101 ± 15% vs. 83 ± 18%, *P* < 0.01;FEV1:103 ± 15% vs. 82 ± 19%, *P* < 0.01;FEF25–75: 118 ± 36% vs. 86 ± 36%, *P* < 0.01; control vs. HF). Airway luminal areas, but not CT measures, were correlated with PFTs at baseline. The SABA cleared EVLW and decreased airway wall thickness but did not change luminal area. Patients with HF had evidence of increased EVLW, but not an expanded bronchial circulation. Airway caliber was maintained relative to controls, despite reductions in lung volume and flow rates. SABA improved lung function, primarily by reducing EVLW.

## Introduction

The heart and lungs are intimately linked, with the disease pathophysiology of one organ system often influencing the other. In heart failure (HF) altered cardiac hemodynamics lead to increased pressure within the lung vasculature contributing to bronchial circulation engorgement and/or a rise in extravascular lung water (EVLW) via increased hydrostatic forces. Concurrently cardiomegaly in HF patients competes with the lungs for space within the thoracic cavity. Reflex‐mediated changes (e.g., due to cardiac stretch), biochemical modulators (e.g., natriuretic peptides, angiotensin II), and alterations in receptors (e.g., beta receptors) all potentially impact airway and vascular function of the lungs. HF patients demonstrate both restrictive and obstructive changes in lung function, but there is substantial heterogeneity in the impact of HF on the pulmonary system (Gehlbach and Geppert 2004). This loss of pulmonary function generally manifests as a decrease in forced vital capacity (FVC) and forced expiratory volume in 1 sec (FEV_1_) as well as other measures of maximal expiratory flow (Ravenscraft et al. 1993; Dimopoulou et al. 1998). These changes in lung function have in turn been shown to contribute to abnormal gas exchange responses, both at rest and during exercise, and subsequently contribute to the symptoms of dyspnea and exercise intolerance, common in this patient population (Johnson 2000).

While previous work has documented specific functional changes to the lungs of HF patients, including decreases in lung volumes and air flows, little work has been done to understand the factors associated with pulmonary congestion and how this in turn alters lung structure and function, especially in more stable disease (Snyder et al. 2006b; Olson et al. 2007). While an increase in EVLW may occur and be causative, swelling of the bronchial circulation may also occur leading to thickening of the airway walls and narrowing of the airway lumen causing reduced function (Cabanes et al. 1989; Ceridon et al. 2009). Previous work has suggested a relationship between the airway luminal area of large to medium‐sized bronchioles and FEV_1_ in healthy individuals; however, the relationship between structural changes and function in the HF population remains unclear (Coxson et al. 2008).

An important modulator of airway caliber and lung fluid regulation in HF patients is the beta 2 adrenergic receptors (ADRB2). These receptors can be desensitized with chronic elevations in catecholamines or chronic use of a long‐acting ADRB2 agonist as seen in asthmatics, though it remains less clear if this occurs in HF (Eaton et al. 2004; Gehlbach and Geppert 2004). In a previous study from our laboratory, we demonstrated that HF patients may have chronic overstimulation of the sympathetic nervous system with evidence for decreased ADRB2 density resulting in altered airway function and reduced lung fluid clearance (Snyder et al. 2006a,b). Stimulating these receptors may lead to improved lymphatic dilation and EVLW clearance as well as a mild influence on airway tone.

The purpose of this study was to determine if HF in stable patients is associated with evidence of chronic congestion and how this in turn might influence airway structure and function in stable HF patients. In addition, we sought to determine if an acutely inhaled ADRB2 agonist would reduce “congestion” and subsequently improve the structure–function relationships. We hypothesized that HF patients would demonstrate swollen airway walls, increased EVLW, or both leading to decreased airway luminal area as compared to healthy controls, particularly in airway generations further from the trachea, leading to the reduction in lung function commonly observed in this population. We also hypothesized acute inhalation of an ADRB2 agonist would reduce swelling of the airway wall as well as EVLW and thus increase airway luminal size and subsequently improve airway function.

## Methods

### Participants

Seventy‐one subjects were recruited for the study; however, the full dataset was not obtained on 11 subjects due to scanner availability. Thirty‐one subjects with a history of HF and 29 age‐ and sex‐matched controls completed all portions of the study. Heart failure subjects had greater than a 1 year history of disease, left ventricular ejection (LVEF) fraction <40%, New York Heart Association (NYHA) functional class of I, II, or III, and a body mass index (BMI) less than 36 kg/m^2^. Subjects were chosen with a range of clinical severity and either nonischemic or ischemic etiology to observe a spectrum of disease. Control subjects had no history of cardiovascular or pulmonary disease and were current nonsmokers with no or minimal smoking history (<15 pack years). The study protocol was approved by Mayo Clinic institutional review board and all subjects provided written informed consent prior to participation.

### Overview of experimental procedures

Experimental procedures were conducted on a single visit day. A complete blood count was assessed to rule out anemia. Spirometric measurements including FVC, FEV_1_, peak expiratory flow (PEF), mean forced expiratory flow between 25% and 75% of the FVC (FEF_25–75_) were assessed according to standard techniques (Miller et al. 2005). Total lung capacity (TLC) was calculated using helium dilution, and a thoracic CT scan was obtained (see below details) (Brown et al. 1998). Albuterol was administered through a nebulizer at a dilution of 2.5 mg per 3 mL of saline over a 12–15 min period. Following albuterol administration, pulmonary function, TLC, and a second CT scan were obtained 45–60 min after nebulization.

### CT scanning, tissue and air volumes, airway segmentation

All CT scans were performed on the same scanner (GE LiteSpeed Spiral CT Scanner, GE Healthcare) and obtained using 2.5‐mm‐thick slices with 1.2 mm overlap and reconstructed to 1.25‐mm‐thick slices with a 0.6 mm overlap. An initial scout scan was performed to ensure capture of the entire lung volume. The location of the scanner table, field of view, and number images taken were recorded. A mark was made on the subject to ensure alignment between pre and postalbuterol scans. Subjects were instructed to hold their breath at TLC during all scans.

Computed tomography quantitative analysis was carried out using MATLAB (Mathworks, Inc, Natick, MA) software. The lung tissue was automatically segmented out from the surrounding tissue using built‐in algorithms. Pixels outside the range of −1000 to 0 HU and airways were excluded from analysis. Values for the mean, skewness, and kurtosis of the distributions were calculated from the segmented areas (Best et al. 2003). Finally, the ratio of pixels in the range greater than −500 HU was calculated as an index of pulmonary congestion (Kato et al. 1996).

Scans were also submitted to image analysis software (Vida Diagnostics, Coralville, IA) for automated analysis. First, the software quantified the volumes of tissue (Vtis) and air (Vair) within the lungs. The fraction of each voxel that represents air and tissue was calculated from the linear attenuation of CT density from −1000 Hounsfield units (Hu) to +55 Hu (Hoffman et al. 2006). The average fraction of air and tissue of each voxel is then multiplied by the total volume of the lung to calculate Vair and Vtis, respectively.

The software used a novel segmentation algorithm that has been described elsewhere to allow segmentation further down the airway tree without “segmentation leak” (Tschirren et al. 2005). The software then calculated the area of the airway and thickness of the airway walls for each generation. The algorithm successfully segmented at least 6 generations from the trachea in all subjects and thus additional generations were dropped from analysis. Where specified, the airway areas were normalized to the subject's TLC, and the airway wall thickness was normalized to the airway area at the same generation to account for differences in body size and to show the relative change with respect to lung volume changes.

### Statistical analysis

All statistical analysis was carried out using SPSS (SPSS, Chicago, IL). The independent sample *t*‐test was used to compare subject characteristics, pulmonary function, and CT‐derived data between HF and healthy populations. The paired sample *t*‐test was used to compare pre to postalbuterol data. A Bonferroni correction was applied for statistical tests across generations. Linear regression and the Pearson correlation coefficient was used to assess the relationship between pulmonary function and lung structure data. All results are expressed as mean ± SD unless otherwise stated. The acceptable level of type I error was defined as *P* < 0.05.

## Results

### Subject demographics

Seventy‐one subjects were recruited for the study. A postalbuterol CT scan could not be obtained in 11 (3 control, 8 HF) subjects due to scanner availability. A complete dataset was obtained for 31 HF patients and 29 age‐matched controls. Subject demographics are shown in Table 1. The two groups were well matched for age and height; however, average weight, BMI, and body surface area (BSA) were higher in the HF group compared to the control subjects (*P* < 0.01). HF patients included those with nonischemic and ischemic etiologies with a range of clinical severities.

**Table 1:** Participant characteristics in heart failure and control subjects. Data are mean plus or minus standard deviation

	Control X ± SD	Heart failure X ± SD
N (Female)	29 (11)	31 (7)
Age, years	56.2 ± 11.5	62.1 ± 11.0
Height, cm	173.6 ± 8.64	174.9 ± 8.76
Weight, kg	77.4 ± 14.4	91.4 ± 17.7^a^
BMI, kg/m^2^	25.6 ± 4.2	29.8 ± 5.0^a^
BSA, m^2^	1.93 ± 0.21	2.10 ± 0.24^a^
LVEF, %	–	28.2 ± 15.3
Heart failure etiology		
Dilated	–	23
Ischemic	–	15
Idiopathic	–	1
NYHA functional class		
I	–	16
II	–	14
III	–	9

BMI, body mass index; BSA, body surface area; LVEF, left ventricular ejection fraction.

a *P* < 0.05 control versus heart failure.

### Quantitative CT indices of congestion

**Figure 1:**
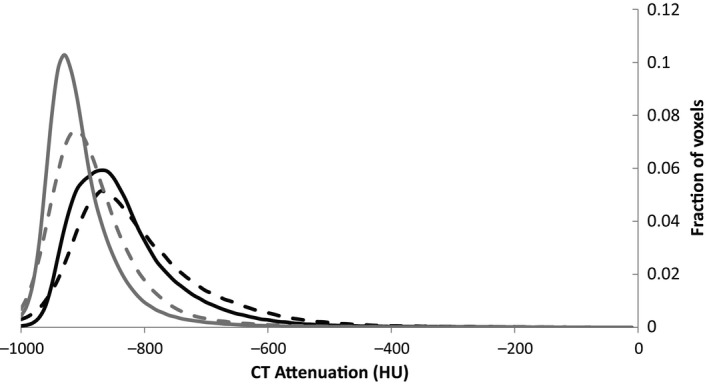
Histogram showing distribution of CT attenuation for the lungs of control (solid line) and heart failure (dashed line) groups at baseline (black) and after albuterol administration (grey). Error bars are not shown for clarity.

The distribution of CT attenuation in the lung is shown in Figure 1. Both groups had a similar mean, but the HF group had a heavier right‐side tail. Thus, the distribution was wider and had a greater percentage of voxels greater than −500 HU in the HF group. Quantitative CT indices are shown in Table 2. The skew, kurtosis, and percentage of voxels greater than −500 HU were significantly different between the HF and control groups (*P* < 0.01) suggesting greater levels of EVLW.

**Table 2:** Baseline values and percent change after albuterol for quantitative CT indices

	Mean (HU)	Skew	Kurtosis	FWHM (HU)	Voxels > −500 HU (%)
Baseline					
Control	−827.4 ± 50.9	2.86 ± 1.07	15.52 ± 9.5	94.5 ± 38.5	1.05 ± 0.83
HF	−804.2 ± 63.9	2.11 ± 0.79^a^	9.34 ± 5.5^a^	131.5 ± 43.7^a^	1.83 ± 1.93^a^
	Mean (%)	Skew (%)	Kurtosis (%)	FWHM (%)	Voxels > −500 HU (%)
Change with Albuterol					
Control	8.49 ± 7.54^b^	54.7 ± 66.9^b^	153.9 ± 194.6^b^	−27.1 ± 22.1^b^	−92.8 ± 7.47^b^
HF	9.27 ± 8.10^b^	86.7 ± 116.1^b^	217.7 ± 290.1^b^	−29.9 ± 18.9^b^	−94.3 ± 5.53^b^

FWHM, full width half max.

a *P* < 0.05 Heart failure (HF) mean versus control mean.

b *P* < 0.05 change from baseline mean different from zero.

### Airway wall thickness

**Figure 2:**
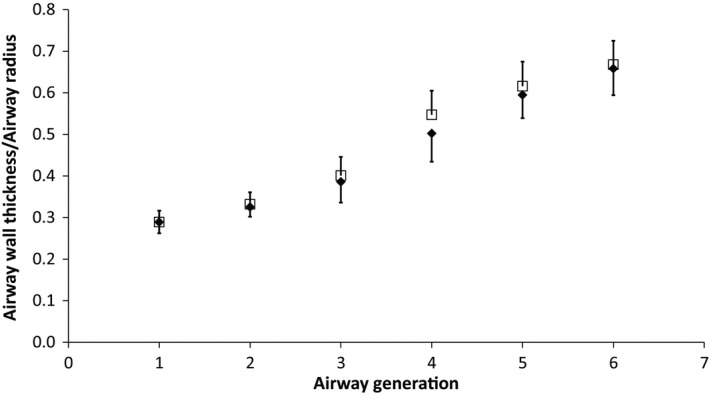
Airway wall thickness normalized to airway radius for the control (solid diamond) and heart failure (open square) population at baseline for airway generations 1 (trachea) to 6. Error bars are standard deviation.

Airway wall thickness decreased by approximately 10% in each successive generation, but there was not a difference between the groups at any generation (healthy, generations 1–6, mm: 2.53, 2.16, 1.71, 1.42, 1.22; HF, generations 1–6, mm: 2.62, 2.21, 2.03, 1.81, 1.48, 1.28; *P* > 0.05). The airway wall thickness as a ratio of the area of that generation increased with increasing generation (Fig. 2). There was no statistical difference between the groups at any generation (*P* > 0.05).

### Airway area

**Figure 3:**
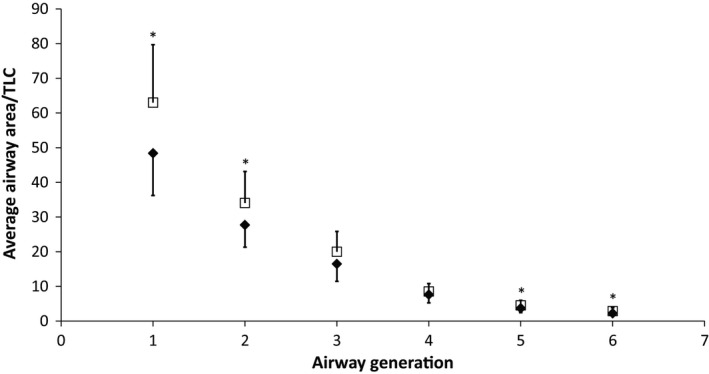
Airway luminal area normalized to TLC for the control (solid diamond) and heart failure (closed square) population at baseline for airway generations 1 (trachea) to 6. Error bars are standard deviation. *P < 0.05 HF mean versus control mean.

Airway area decreased by approximately half at each successive generation and were not significantly different at any generation between groups (healthy, generations 1–6, mm: 245, 141, 83, 39, 19; HF, generations 1–6, mm: 267, 143, 83, 36, 19; *P* > 0.05). Airway area normalized to TLC is shown in Figure 3. The normalized airway area was significantly greater (*P* < 0.01) for all generations except generations 3 and 4 in the HF group.

### Pulmonary function

**Figure 4:**
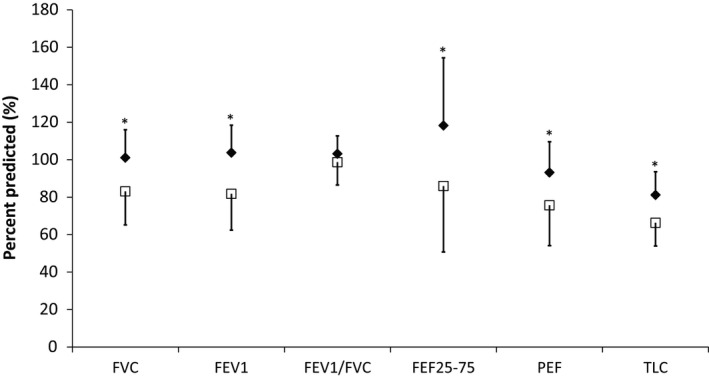
Pulmonary function measurements for the control (solid diamond) and heart failure (open square) population at baseline. Error bars are standard deviation. FVC, forced vital capacity; FEV_1_, forced expiratory volume in 1 sec; FEF_25–75_, forced expiratory volume at 25–75% of FVC; PEF, peak expiratory flow; TLC, total lung capacity. *P < 0.05 HF mean versus control mean.

Pulmonary function characteristics are shown in Figure 4 for both groups. Percent predicted FVC, FEV_1_, forced expiratory flow 25–75% (FEF_25–75_), and peak expiratory flow (PEF) were significantly lower (*P* < 0.01) in the HF group, while the FEV_1_/FVC ratio was not significantly different.

### Structure–function relationships

Linear regression was performed to assess the relationship between airway area and CT quantitative indices and lung function. There were no correlations between CT tissue histogram parameters and pulmonary function at baseline. Pearson correlation coefficients for airway area versus lung function are shown in Table 3. In the control group, generation 2 was significantly correlated with FEV_1_, FVC, FEF_25–75_, and PEF, and PEF was correlated with all generations except generation 3. In the HF group, generations 1, 2, and 4 were significantly correlated with all measures, and PEF was correlated with all generations.

**Table 3:** Pearson correlation coefficients (*r*) for airway area versus pulmonary function at baseline

	Airway Generation					
	1	2	3	4	5	6
Control						
FEV_1_	0.46	0.56^a^	0.32	0.31	0.31	0.29
FVC	0.39	0.53^a^	0.33	0.31	0.28	0.31
FEF_25–75_	0.53^a^	0.49^a^	0.24	0.25	0.40	0.30
PEF	0.59^a^	0.63^a^	0.25	0.40^a^	0.54^a^	0.47^a^
Heart failure						
FEV_1_	0.55^a^	0.49^a^	0.40	0.51^a^	0.44^a^	0.28
FVC	0.54^a^	0.47^a^	0.38	0.47^a^	0.35	0.19
FEF_25–75_	0.50^a^	0.47^a^	0.31	0.46^a^	0.50^a^	0.40
PEF	0.64^a^	0.62^a^	0.58^a^	0.50^a^	0.54^a^	0.46^a^

a *P* < 0.05.

### Effects of an ADRB2 agonist on lung structure and function

**Figure 5:**
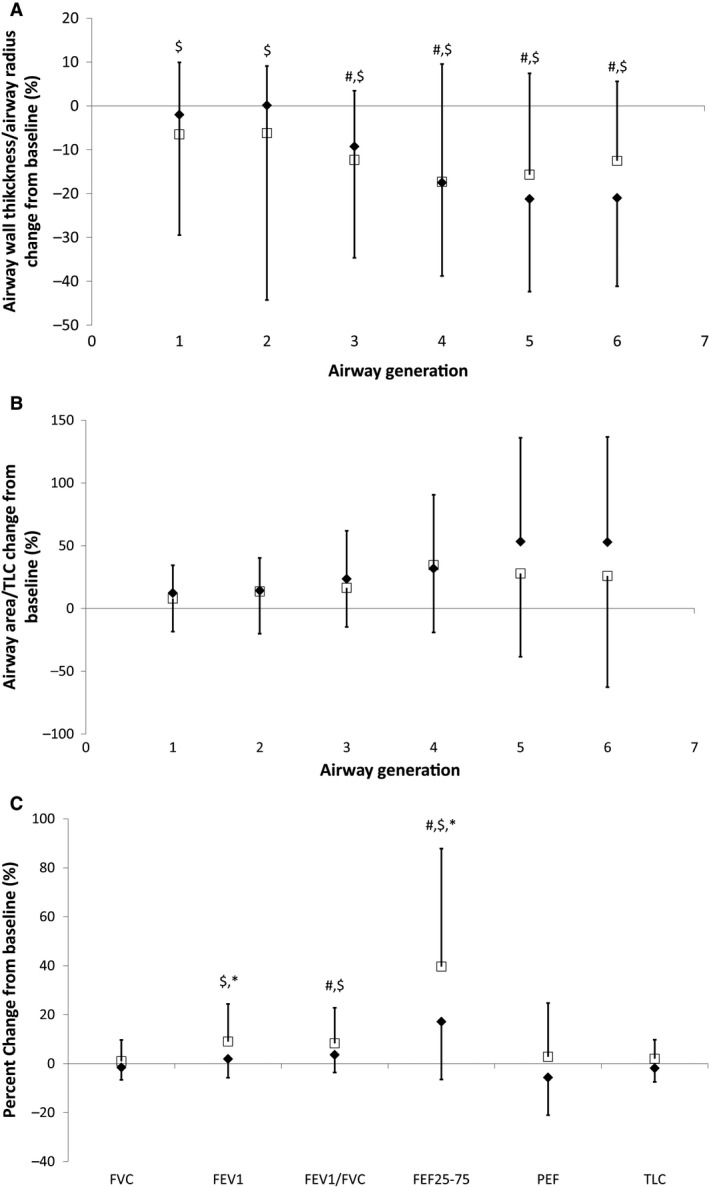
Percent change from baseline after ADRB2 agonist administration for airway wall thickness normalized to airway luminal area (A), airway luminal area normalized to TLC (B), and lung function measures (C) for the control (solid diamond) and heart failure (closed square) groups. Error bars are standard deviation. *P < 0.05 HF mean versus control mean, ^#^ *P* < 0.05 Control change from baseline mean different from zero, ^$^ *P* < 0.05 HF change from baseline mean different from zero.

After ADRB2 agonist administration, the CT attenuation distributions for both groups shifted significantly to the left and were narrower relative to baseline (Fig. 1). The mean, skew, kurtosis, full width half max (FWHM), and percentage of voxels greater than −500 HU changed significantly with similar changes between the groups (*P* < 0.01). Linear regression for baseline values versus percent change of mean attenuation, skew, kurtosis, FWHM, and percentage of voxels greater than −500 HU showed a statistically significant correlation for all values in both groups suggesting greater clearance in those with more fluid at baseline (Table 4). Agonist administration increased the absolute size of the wall and decreased the fraction of the wall relative to the area significantly (*P* < 0.05) in all generations for the HF group and generations 3 through 6 for the control group (Fig. 5A). There was no statistical difference between the groups after agonist administration. After albuterol administration, neither the absolute (not shown) nor the normalized airway areas changed for either group or between groups (Fig. 5B). After albuterol administration (Fig. 5C), FEV_1_, FEV_1_/FVC, and FEF_25–75_ improved significantly for the HF group (*P* < 0.05) and FEV_1_ and FEF_25–75_ improved for the control group (*P* < 0.05). The HF group improved more than the control group for FEV_1_ and FEF_25–75_ (*P* < 0.05). There was no difference after albuterol for FVC, PEF, and TLC between groups. There were no statistically significant correlations between changes in pulmonary function and CT quantitative indices or airway areas.

**Table 4:** Pearson correlation coefficients (*r*) for change in quantitative CT indices at baseline versus change after albuterol for both groups

	Mean	Skew	Kurt	FWHM	Voxels < 500 HU
Control	0.91^a^	−0.71^a^	−0.68^a^	−0.67^a^	−0.50^a^
HF	0.88^a^	−0.80^a^	−0.69^a^	−0.60^a^	−0.57^a^

a *P* < 0.05.

## Discussion

In this study, we examined measures of lung congestion, airway structure and the relationship between airway structure and lung function in HF patients and in control subjects. In addition, we investigated if an acutely inhaled ADRB2 agonist would help clear lung fluid and as a result modulate airway structure–function relationships. We found evidence for increased EVLW in the HF population; however, no evidence was observed of an engorged or swollen bronchial circulation as inferred by CT quantification of airway walls. In addition, we found relatively well preserved airway luminal areas in the first six generations of airways despite significantly reduced lung function. The inhaled ADRB2 agonist shifted the lung density histogram away from water in both HF and control, had no impact on airway wall thickness or airway luminal areas, and improved lung function. Despite the parallel changes in EVLW and lung function there was no clear relationship between measures of air flow or lung volumes and EVLW clearance. While we were not able to detect a relationship, the increased fluid and decreased air flows and volumes suggests that increased lung fluid may be important in stable HF.

### Extravascular lung water

Extravascular lung water (EVLW) is known to alter lung mechanics and could contribute to the loss of function in HF, but it is difficult to quantitatively measure in vivo (Grossman et al. 1980; Esbenshade et al. 1982). Quantitative CT indices, skew and kurtosis of the CT attenuation distribution have previously been used to study interstitial lung disease and pulmonary edema in HF. In patients with idiopathic pulmonary fibrosis, disease progression is associated with increased CT attenuation mean, decreased skew, and decreased kurtosis and statistically significant correlations between mean, kurtosis, and skew with FVC and FEV_1_ (Hartley et al. 1994). In a previous study of HF patients, histogram analysis showed a decreased CT attenuation mean and percent of pixels greater than −500 HU increased for subjects with severe pulmonary congestion (Kato et al. 1996). In this study, we found evidence for more fluid in the lung tissue in HF patients compared to controls despite our patients being stable and optimally managed (Table 2). The CT quantitative indices measure both EVLW and small blood vessels; however, it has been shown that small blood vessel volume is similar in HF patients versus controls, suggesting that these indices are measuring primarily EVLW (Agostoni et al. 2006). Increased EVLW may decrease lung compliance in HF as has been found in previous studies (Frank et al. 1957). Our laboratory has previously found increased elastic work of breathing during exercise, but not at low levels of ventilation, in stable HF patients suggesting decreased lung compliance (Cross et al. 2012). While we found a mild increase in EVLW, we did not find a clear relationship between the quantitative CT indices of EVLW and measures of maximal air flow or lung volumes. One explanation may be that the lung may be able to tolerate a mild level of extravascular fluid accumulation without a direct impact on lung function; however, preventing further increases in EVLW accumulation may be important in preventing decompensation. EVLW tends to flow toward the lymph system and avoid gas exchange areas and the lymphatics in general may be upregulated in the HF population due to a rapid shallow breathing pattern and a rise in circulating catecholamines.

### Airway wall thickness and luminal area

Numerous studies have attempted to understand the factors that contribute to loss of pulmonary function in HF. Heart size has been found to be a significant factor accounting for loss of lung volume (Olson et al. 2007). We hypothesized that airway structure would also change with the development of HF and increased EVLW. Specifically, we hypothesized that HF (associated with increased pulmonary wedge pressure) leading to vascular engorgement of the bronchial circulation within the bronchiole walls would cause thickening of the airway walls and a subsequent decrease in luminal areas. Previous work has suggested that the bronchial circulation is contained within the airway wall, and its modulation can improve exercise capacity in individuals with HF (Cabanes et al. 1989; King et al. 2002). Interestingly, we found that HF patients maintained airway wall thickness and luminal areas similar to control subjects, at least through six airway generations from the trachea (Figs 2 and 3).The lack of change in the wall thickness suggests that the bronchial circulation is not expanded in this population. Previous studies in humans and animals after rapid fluid loading have shown increased wall thicknesses and decreased luminal areas, especially in smaller airways (Michel et al. 1986, 1987; Brown et al. 1995; King et al. 2002). However, one study found changes in respiratory bronchioles and bronchioles, but not in bronchi after rapid fluid loading in dogs, and a study in humans found changes in airway wall thickness and luminal areas in only some large airways after fluid loading healthy subjects (Michel et al. 1986; Ceridon et al. 2010). In this study, we found relatively mild levels of edema, which may not be enough to cause significant swelling of the airway walls or narrowing of the airway luminal area. Additionally, only generations 0 (trachea) through 6 (mid‐sized bronchi) were examined, which are relatively large, primarily cartilaginous airways whose walls may be stiff enough to resist engorgement with increased vascular volumes or compression of the airways from increased interstitial fluid (Voets and van Helvoort 2013).

### Airway structure and lung function

Pulmonary function exhibited both restrictive and obstructive changes in HF patients in this study (Fig. 4). Previous studies have shown approximately 20% reductions in FVC, FEV_1_, and FEF_25–75_ with the development of HF (Snyder et al. 2006b; Lizak et al. 2009). However, in both the control and HF groups, the airway luminal area in at least one generation was significantly related to each of the four spirometry variables, FEV_1_, FVC, FEF_25–75_, and PEF at baseline. It is possible that the airway generations most responsible for modulating lung function with disease and after ADRB2 agonist administration are located below the resolution of CT scanning (beyond generation 6); this is discussed in more depth below (see *Limitations*). Furthermore, the location of the equal pressure point, or the point of airway narrowing or collapse during a maximal expiration, is dependent on the resistance and luminal area of the airways leading to limitations of flow through that segment independent of driving pressure (Mead et al. 1967; Voets and van Helvoort 2013). This study suggests that it is necessary to quantify smaller airways to properly characterize the relationship between airway structure and function.

### Effect of an ADRB2 agonist

Albuterol, an ADRB2 agonist, has been shown to bronchodilate and clear EVLW through alveolar and lymphatic mechanisms (Lauweryns and Baert 1977; Giembycz and Raeburn 1991; Eaton et al. 2004). Lymphatic fluid clearance has been shown to rapidly increase after pharmacological stimulation and in response to exercise in animal models (Coates et al. 1984; Frank et al. 2000). Albuterol effectively cleared fluid from both populations and cleared more fluid in those subjects with more fluid at baseline (Table 4). There was an effect of decreasing the size of the airway wall relative to the luminal area but no effect on the size of the airways for generations that could be observed using CT imaging (Fig. 6A and B). Albuterol administration improved air flows (FEV_1_ and FEF_25–75_), but not lung volumes, in both groups (Fig. 6C). This improvement in FEF_25–75_ suggests that the effects of albuterol may be on smaller airways than those observed here. We did not find statistically significant correlations between changes in airway structure and lung function from before to after ADBR2 administration. However, these findings are similar to those found in study involving fluid loading healthy subjects. Fluid loading decreased pulmonary function and changes were noted in airway luminal area and wall thickness; however, relationships were not found between the changes in airway structure measurements of these large airways and lung function measurements (Ceridon et al. 2010). While ADRB2 agonists have been historically considered contraindicated in HF, inhaled agonists have not been shown to increase dysrhythmias (Maak et al. 2011).Therefore, an ADRB2 agonist may be an effective method of improving lung function and clearing lung fluid in volume overloaded HF patients.

### Limitations

There are four major limitations of this study. First, the difference in weight between the two groups may have affected the differences in PFTs. However, one study showed that increased body weight is associated with increased spirometric parameters, suggesting that the differences observed are not a result of body weight differences (Omori et al. 2014). Second, all CT measurements were taken at TLC, which may not be representative of functional lung volumes. A previous study in individuals with asthma suggests that lung inflation to TLC can affect the area of the large airways measured here (Brown et al. 2001). However, to limit radiation only one CT image was taken per treatment condition. We chose to measure at TLC to maximize the size of the airways and the ability of the software to properly segment the airway structures(Brown et al. 2001). Third, the resolution of the CT scanning may not be sufficient to detect changes at smaller airway generations. At generation 6, the diameter of the airway is approximately 2 pixels, making the measurement of airway area and luminal area susceptible to partial volume effects. Nevertheless, this study has characterized the more proximal airways that tend to be cartilaginous, which are still an important determinant of maximal air flow (Coxson et al. 2008). Finally, the sensitivity of the automatic airway segmentation algorithm may not properly segment all possible airways. While the automatic segmentation algorithm has been well validated on healthy individuals, it may occasionally miss airway segments or, alternatively, segment nonairway structures, leading to large standard deviations in the measurement of airway area and wall thickness. In order to account for these potential occurrences, we have been careful to visually validate the automatic segmentation to remove significant deviations from structures of interest.

## Conclusion

The heart and lungs are intimately linked and during the development and progression of the HF syndrome the pulmonary system undergoes significant changes that in turn contribute to the pathophysiology of the disease through alterations in lung function, breathing pattern, respiratory gas exchange, and ultimately symptomatology. This study examined the structure–function relationships of the pulmonary system in HF patients and the influence of an acutely inhaled ADRB2 agonist; known to dilate airways and stimulate extravascular lung fluid clearance. We determined that EVLW was increased in clinically stable HF patients. However, airway wall thicknesses and airway luminal areas were maintained relative to healthy controls in the large airways studied, despite significant reductions in pulmonary function. An acutely nebulized ADRB2 agonist caused significant clearance of EVLW, but did not change airway wall thickness or luminal area in the large generations, suggesting the importance of lung fluid in stable HF patients, and the possibility of ADRB2 agonists as a treatment in improving lung air flows and volumes and clearing EVLW.

## Conflict of Interest

None declared.

## References

[phy212867-bib-0001] Agostoni, P. , M. Bussotti , G. Cattadori , E. Margutti , M. Contini , M. Muratori , et al. 2006 Gas diffusion and alveolar‐capillary unit in chronic heart failure. Eur. Heart J. 27:2538–2543. 17028107 10.1093/eurheartj/ehl302

[phy212867-bib-0002] Best, A. C. , A. M. Lynch , C. M. Bozic , D. Miller , G. K. Grunwald , and D. A. Lynch . 2003 Quantitative CT indexes in idiopathic pulmonary fibrosis: relationship with physiologic impairment. Radiology 228:407–414. 12802000 10.1148/radiol.2282020274

[phy212867-bib-0003] Brown, R. H. , E. A. Zerhouni , and W. Mitzner . 1995 Airway edema potentiates airway reactivity. J. Appl. Physiol. (1985) 79:1242–1248. 8567568 10.1152/jappl.1995.79.4.1242

[phy212867-bib-0004] Brown, R. , D. E. Leith , and P. L. Enright . 1998 Multiple breath helium dilution measurement of lung volumes in adults. Eur. Respir. J. 11:246–255. 9543301 10.1183/09031936.98.11010246

[phy212867-bib-0005] Brown, R. H. , N. Scichilone , B. Mudge , F. B. Diemer , S. Permutt , and A. Togias . 2001 High‐resolution computed tomographic evaluation of airway distensibility and the effects of lung inflation on airway caliber in healthy subjects and individuals with asthma. Am. J. Respir. Crit. Care Med. 163:994–1001. 11282779 10.1164/ajrccm.163.4.2007119

[phy212867-bib-0006] Cabanes, L. R. , S. N. Weber , R. Matran , J. Regnard , M. O. Richard , M. E. Degeorges , et al. 1989 Bronchial hyperresponsiveness to methacholine in patients with impaired left ventricular function. N. Engl. J. Med. 320:1317–1322. 2541334 10.1056/NEJM198905183202005

[phy212867-bib-0007] Ceridon, M. , A. Wanner , and B. D. Johnson . 2009 Does the bronchial circulation contribute to congestion in heart failure? Med. Hypotheses 73:414–419. 19464810 10.1016/j.mehy.2009.03.033 PMC2738626

[phy212867-bib-0008] Ceridon, M. L. , E. M. Snyder , N. A. Strom , J. Tschirren , and B. D. Johnson . 2010 Influence of rapid fluid loading on airway structure and function in healthy humans. J. Card. Fail. 16:175–185. 20142030 10.1016/j.cardfail.2009.08.005 PMC2885053

[phy212867-bib-0009] Coates, G. , H. O'Brodovich , A. L. Jefferies , and G. W. Gray . 1984 Effects of exercise on lung lymph flow in sheep and goats during normoxia and hypoxia. J. Clin. Invest. 74:133–141. 6736245 10.1172/JCI111393 PMC425193

[phy212867-bib-0010] Coxson, H. O. , B. Quiney , D. D. Sin , L. Xing , A. M. McWilliams , J. R. Mayo , et al. 2008 Airway wall thickness assessed using computed tomography and optical coherence tomography. Am. J. Respir. Crit. Care Med. 177:1201–1206. 18310475 10.1164/rccm.200712-1776OC PMC2408438

[phy212867-bib-0011] Cross, T. J. , S. Sabapathy , K. C. Beck , N. R. Morris , and B. D. Johnson . 2012 The resistive and elastic work of breathing during exercise in patients with chronic heart failure. Eur. Respir. J. 39:1449–1457. 22034652 10.1183/09031936.00125011 PMC3951372

[phy212867-bib-0012] Dimopoulou, I. , M. Daganou , O. K. Tsintzas , and G. E. Tzelepis . 1998 Effects of severity of long‐standing congestive heart failure on pulmonary function. Respir. Med. 92:1321–1325. 10197224 10.1016/s0954-6111(98)90136-6

[phy212867-bib-0013] Eaton, D. C. , J. Chen , S. Ramosevac , S. Matalon , and L. Jain . 2004 Regulation of Na+ channels in lung alveolar type II epithelial cells. Proc. Am. Thorac. Soc. 1:10–16. 16113405 10.1513/pats.2306008

[phy212867-bib-0014] Esbenshade, A. M. , J. H. Newman , P. M. Lams , H. Jolles , and K. L. Brigham . 1982 Respiratory failure after endotoxin infusion in sheep: lung mechanics and lung fluid balance. J. Appl. Physiol. Respir. Environ. Exerc. Physiol. 53:967–976. 6759492 10.1152/jappl.1982.53.4.967

[phy212867-bib-0015] Frank, N. R. , H. A. Lyons , A. A. Siebens , and T. F. Nealon . 1957 Pulmonary compliance in patients with cardiac disease. Am. J. Med. 22:516–523. 13410946 10.1016/0002-9343(57)90106-7

[phy212867-bib-0016] Frank, J. A. , Y. Wang , O. Osorio , and M. A. Matthay . 2000 Beta‐adrenergic agonist therapy accelerates the resolution of hydrostatic pulmonary edema in sheep and rats. J. Appl. Physiol. (1985) 89:1255–1265. 11007557 10.1152/jappl.2000.89.4.1255

[phy212867-bib-0017] Gehlbach, B. K. , and E. Geppert . 2004 The pulmonary manifestations of left heart failure. Chest 125:669–682. 14769751 10.1378/chest.125.2.669

[phy212867-bib-0018] Giembycz, M. A. , and D. Raeburn . 1991 Putative substrates for cyclic nucleotide‐dependent protein kinases and the control of airway smooth muscle tone. J. Auton. Pharmacol. 11:365–398. 1662219 10.1111/j.1474-8673.1991.tb00260.x

[phy212867-bib-0019] Grossman, R. F. , J. G. Jones , and J. F. Murray . 1980 Effects of oleic acid‐induced pulmonary edema on lung mechanics. J. Appl. Physiol. Respir. Environ. Exerc. Physiol. 48:1045–1051. 7380701 10.1152/jappl.1980.48.6.1045

[phy212867-bib-0020] Hartley, P. G. , J. R. Galvin , G. W. Hunninghake , J. A. Merchant , S. J. Yagla , S. B. Speakman , et al. 1994 High‐resolution CT‐derived measures of lung density are valid indexes of interstitial lung disease. J. Appl. Physiol. (1985) 76:271–277. 8175517 10.1152/jappl.1994.76.1.271

[phy212867-bib-0021] Hoffman, E. A. , B. A. Simon , and G. McLennan . 2006 State of the Art. A structural and functional assessment of the lung via multidetector‐row computed tomography: phenotyping chronic obstructive pulmonary disease. Proc. Am. Thorac. Soc. 3:519–532. 16921136 10.1513/pats.200603-086MS PMC2647643

[phy212867-bib-0022] Johnson, R. L. Jr . 2000 Gas exchange efficiency in congestive heart failure. Circulation 101:2774–2776. 10859280 10.1161/01.cir.101.24.2774

[phy212867-bib-0023] Kato, S. , T. Nakamoto , and M. Iizuka . 1996 Early diagnosis and estimation of pulmonary congestion and edema in patients with left‐sided heart diseases from histogram of pulmonary CT number. Chest 109:1439–1445. 8769490 10.1378/chest.109.6.1439

[phy212867-bib-0024] King, L. S. , S. Nielsen , P. Agre , and R. H. Brown . 2002 Decreased pulmonary vascular permeability in aquaporin‐1‐null humans. Proc. Natl. Acad. Sci. U. S. A. 99:1059–1063. 11773634 10.1073/pnas.022626499 PMC117429

[phy212867-bib-0025] Lauweryns, J. M. , and J. H. Baert . 1977 Alveolar clearance and the role of the pulmonary lymphatics. Am. Rev. Respir. Dis. 115:625–683. 322558 10.1164/arrd.1977.115.4.625

[phy212867-bib-0026] Lizak, M. K. , M. Zakliczynski , A. Jarosz , and M. Zembala . 2009 The influence of chronic heart failure on pulmonary function tests in patients undergoing orthotopic heart transplantation. Transplant Proc. 41:3194–3197. 19857708 10.1016/j.transproceed.2009.07.072

[phy212867-bib-0027] Maak, C. A. , J. A. Tabas , and D. E. McClintock . 2011 Should acute treatment with inhaled beta agonists be withheld from patients with dyspnea who may have heart failure? J. Emerg. Med. 40:135–145. 18572345 10.1016/j.jemermed.2007.11.056

[phy212867-bib-0028] Mead, J. , J. M. Turner , P. T. Macklem , and J. B. Little . 1967 Significance of the relationship between lung recoil and maximum expiratory flow. J. Appl. Physiol. 22:95–108. 6017658 10.1152/jappl.1967.22.1.95

[phy212867-bib-0029] Michel, R. P. , S. Meterissian , and R. S. Poulsen . 1986 Morphometry of the distribution of hydrostatic pulmonary oedema in dogs. Br. J. Exp. Pathol. 67:865–877. 3801300 PMC2013123

[phy212867-bib-0030] Michel, R. P. , L. Zocchi , A. Rossi , G. A. Cardinal , Y. Ploy‐Song‐Sang , R. S. Poulsen , et al. 1987 Does interstitial lung edema compress airways and arteries? A morphometric study. J. Appl. Physiol. (1985) 62:108–115. 3104281 10.1152/jappl.1987.62.1.108

[phy212867-bib-0031] Miller, M. R. , J. Hankinson , V. Brusasco , F. Burgos , R. Casaburi , A. Coates , et al. 2005 Standardisation of spirometry. Eur. Respir. J. 26:319–338. 16055882 10.1183/09031936.05.00034805

[phy212867-bib-0032] Olson, T. P. , K. C. Beck , and B. D. Johnson . 2007 Pulmonary function changes associated with cardiomegaly in chronic heart failure. J. Card. Fail. 13:100–107. 17395049 10.1016/j.cardfail.2006.10.018 PMC1941841

[phy212867-bib-0033] Omori, H. , A. Onoue , T. Katoh , Y. Ogata , H. Kawashima , N. Miyao , et al. 2014 A large cohort study concerning age‐dependent impacts of anthropometric variables on spirometric parameters in nonsmoking healthy adults. PLoS ONE 9:e100733. 24955585 10.1371/journal.pone.0100733 PMC4067384

[phy212867-bib-0034] Ravenscraft, S. A. , C. R. Gross , S. H. Kubo , M. T. Olivari , S. J. Shumway , R. M. Bolman 3rd , et al. 1993 Pulmonary function after successful heart transplantation One year follow‐up. Chest 103:54–58. 8417937 10.1378/chest.103.1.54

[phy212867-bib-0035] Snyder, E. M. , M. L. Hulsebus , S. T. Turner , M. J. Joyner , and B. D. Johnson . 2006a Genotype related differences in beta2 adrenergic receptor density and cardiac function. Med. Sci. Sports Exerc. 38:882–886. 16672841 10.1249/01.mss.0000218144.02831.f6

[phy212867-bib-0036] Snyder, E. M. , S. T. Turner , and B. D. Johnson . 2006b Beta2‐adrenergic receptor genotype and pulmonary function in patients with heart failure. Chest 130:1527–1534. 17099033 10.1378/chest.130.5.1527

[phy212867-bib-0037] Tschirren, J. , E. A. Hoffman , G. McLennan , and M. Sonka . 2005 Intrathoracic airway trees: segmentation and airway morphology analysis from low‐dose CT scans. IEEE Trans. Med. Imaging 24:1529–1539. 16353370 10.1109/TMI.2005.857654 PMC1851666

[phy212867-bib-0038] Voets, P. J. , and H. A. van Helvoort . 2013 The role of equal pressure points in understanding pulmonary diseases. Adv. Physiol. Educ. 37:266–267. 24022774 10.1152/advan.00014.2013

